# Supplementary material for: Gut micro-organisms associated with health, nutrition and dietary interventions
Source: Nature. 2025 Dec 10;650(8101):450–8. doi: 10.1038/s41586-025-09854-7 (PMC12893911; doi:10.1038/s41586-025-09854-7)
Supplement: Supplementary file 1 — Supplementary Figs. 1–5 and table legends. [file 41586_2025_9854_MOESM1_ESM.pdf]

nature portfolio

https://doi.org/10.1038/s41586-025-09854-7

Supplementary information

Gut micro-organisms associated with health, nutrition and dietary interventions

In the format provided by the
authors and unedited

Gut microbes associated with health, nutrition and dietary interventions

Francesco Asnicar [1,^], Paolo Manghi [1,+], Gloria Fackelmann [1], Gabriel Baldanzi [1], Elco Bakker [2], Liviana Ricci [1], Gianmarco Piccinno [1], Elisa Piperni [1,3], Katarina Mladenovic [1], Federica Amati [2,4], Alberto Arrè [2], Sajaysurya Ganesh [2], Francesca Giordano [2], Richard Davies [2], Jonathan Wolf [2], Kate M. Bermingham [2,5], Sarah E. Berry [5,2,*], Tim D. Spector [4,2,*], Nicola Segata [1,3,4,*,^]

^ correspondence: f.asnicar@unitn.it, nicola.segata@unitn.it

Supplementary Information Guide

Table of Contents

Supplementary Fig. 1 2
Supplementary Fig. 2 3
Supplementary Fig. 3 4
Supplementary Fig. 4 5
Supplementary Fig. 5 7
Supplementary Table Legends 8

Supplementary Fig. 1

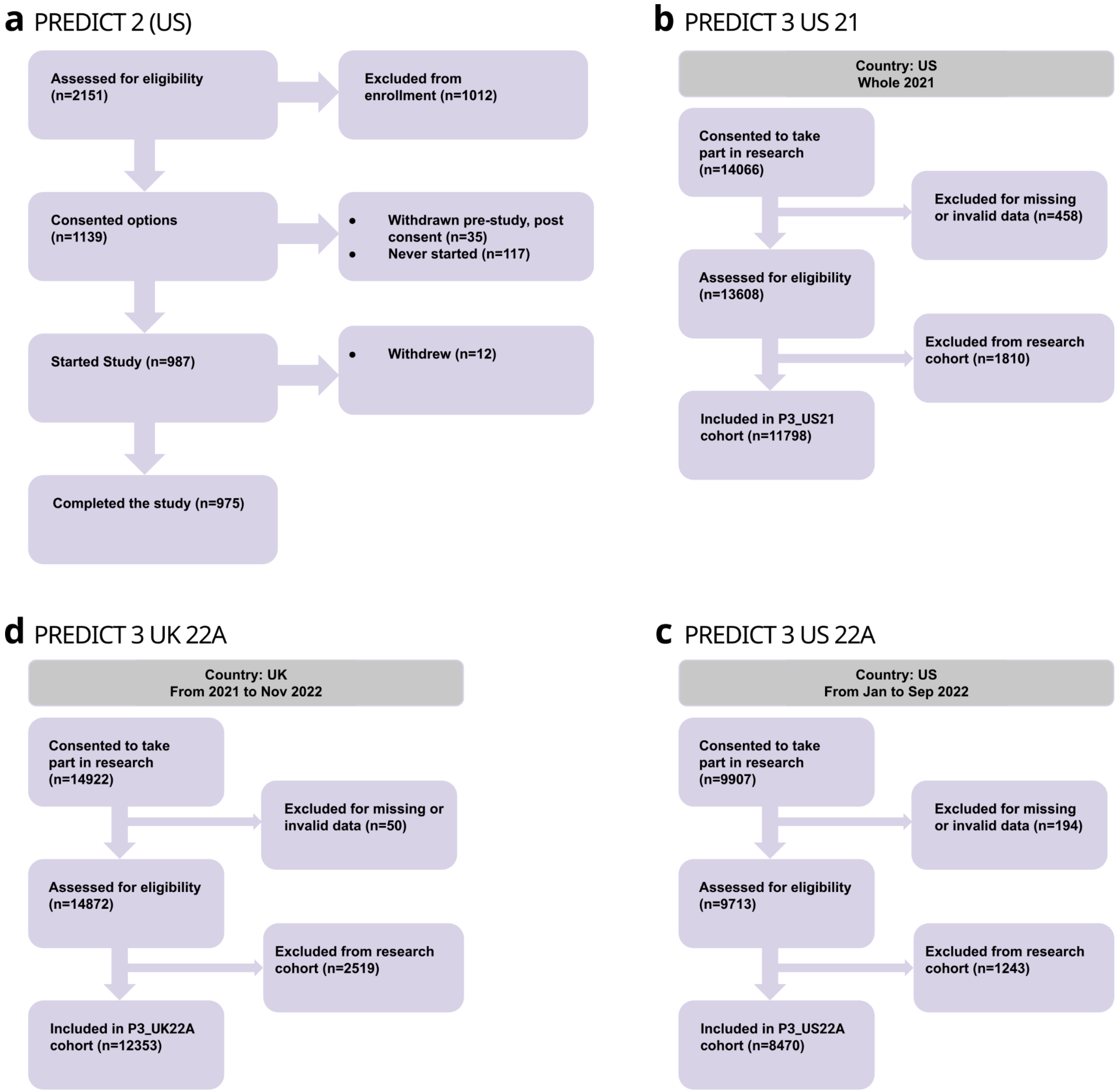

Supplementary Fig. 1. CONSORT diagrams for the ZOE cohorts. Description of the starting number of enrolled individuals in each of the new ZOE cohorts, with the details of the exclusion criteria and the number of individuals removed at each step. **a)** The PREDICT 2 cohort comprised a total of 975 individuals from the US (NCT03983733). **b-e)** The PREDICT 3 cohorts (ClinicalTrials.gov ID: NCT04735835) are composed of ZOE customers who consented for their microbiome data to be used for research purposes. **b)** The PREDICT 3 US21 cohort comprised a total of 11,798 individuals from the US. **c)** The PREDICT 3 US22A cohort comprised a total of 8,470 individuals from the US. **d)** The PREDICT 3 UK22A cohort comprised a total of 12,353 individuals from the UK.

Supplementary Fig. 2

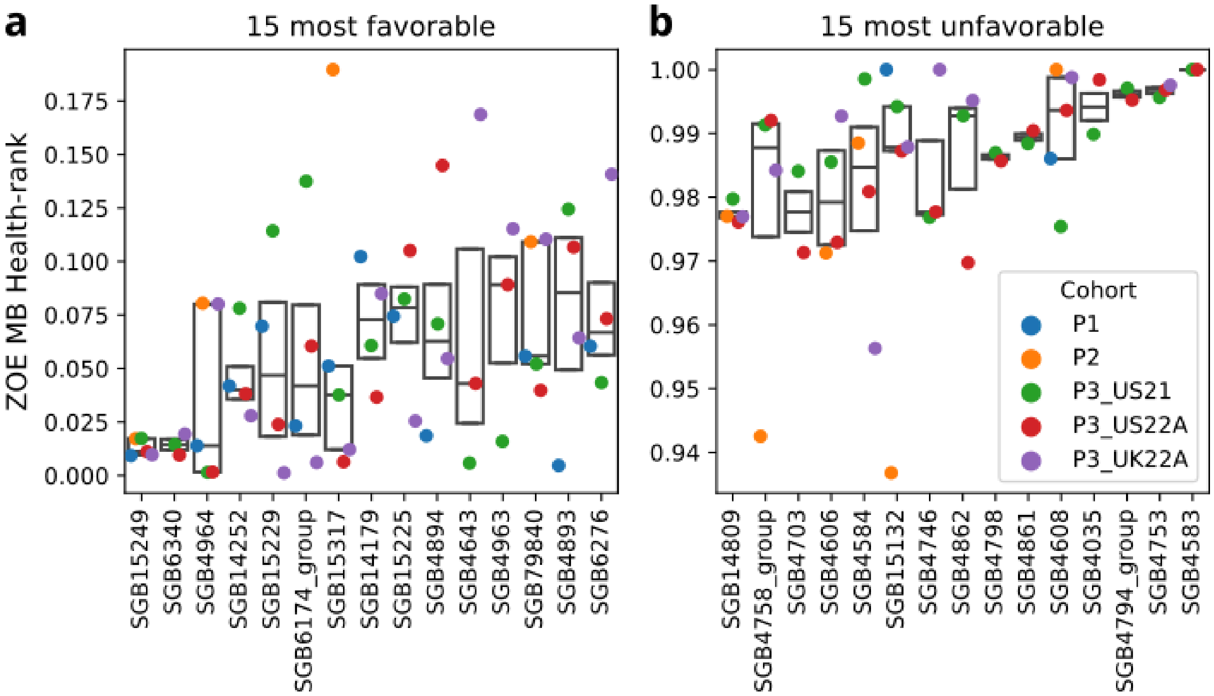

Supplementary Fig. 2. Cohort-specific distribution of ranks for the 15 most favorably and unfavorably ZME MB Health-ranked SGBs. Boxplots show the rank in the single PREDICT cohort of the fifteen most favorable (**a**) and unfavorable (**b**) ZOE MB Health-ranked SGBs (**Methods**). **a)** The 15 most favorably ranked SGBs according to the ZOE MB Health-ranks, show rank values in each PREDICT cohort consistently close to zero. **b)** On the contrary, the 15 most unfavorably ranked SGBs according to the ZOE MB Health-ranks, generally show rank values close to one in all cohorts.

Supplementary Fig. 3

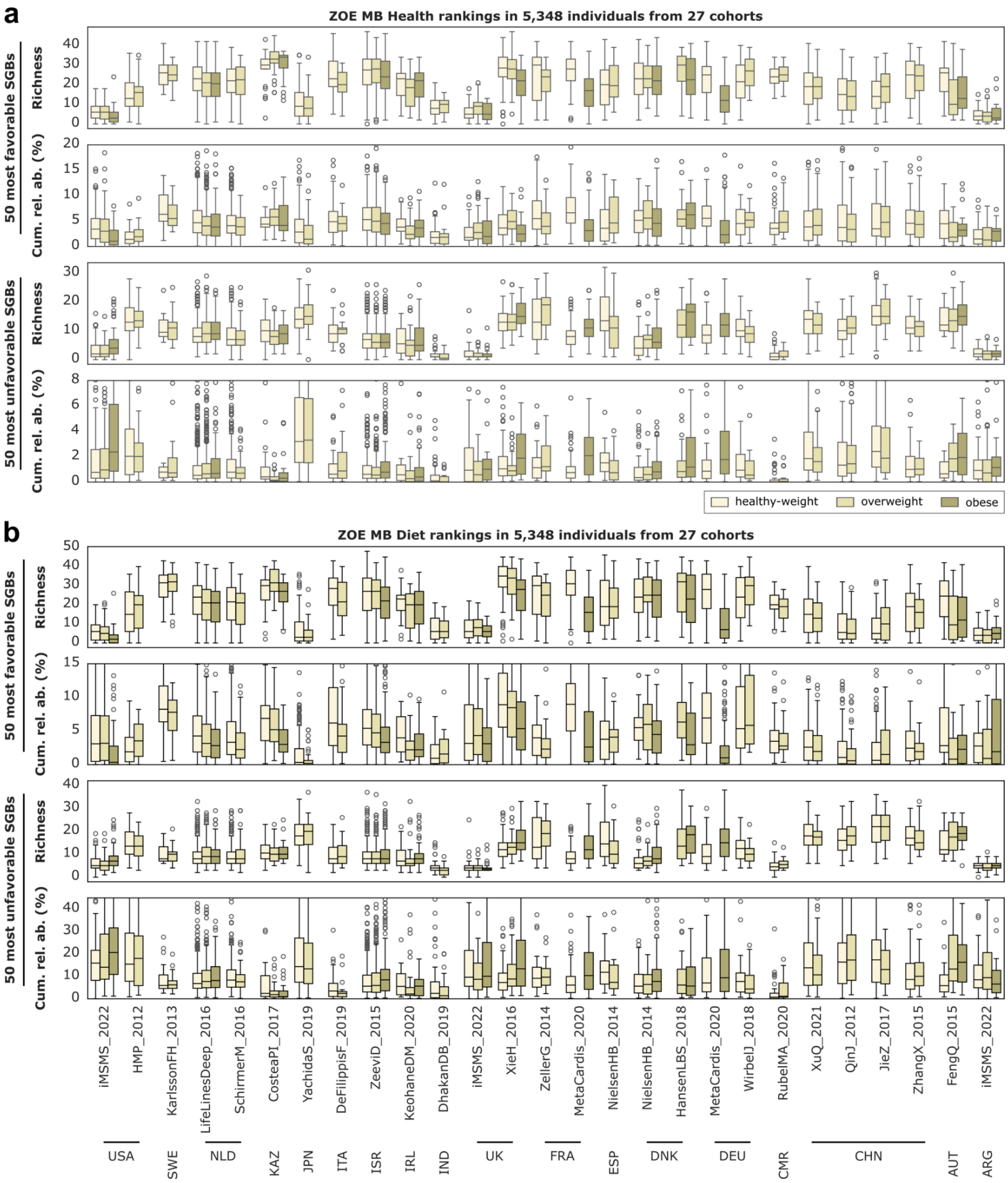

Supplementary Fig. 3. The ZOE MB Health and Diet-ranked SGBs stratify individuals according to their BMI in 27 public cohorts. The number and cumulative relative abundance of the 50 most favorably ranked SGBs from **a)** the ZOE MB Health rank and **b)** the ZOE MB Diet rank detected in individuals from 27 public datasets, showed that increasing BMI is reflected by a lower presence of favorable SGBs (top two rows). On the other hand, the 50 most unfavorably-ranked SGBs show an increasing count and cumulative abundance in higher BMI categories (bottom two rows).

Supplementary Fig. 4

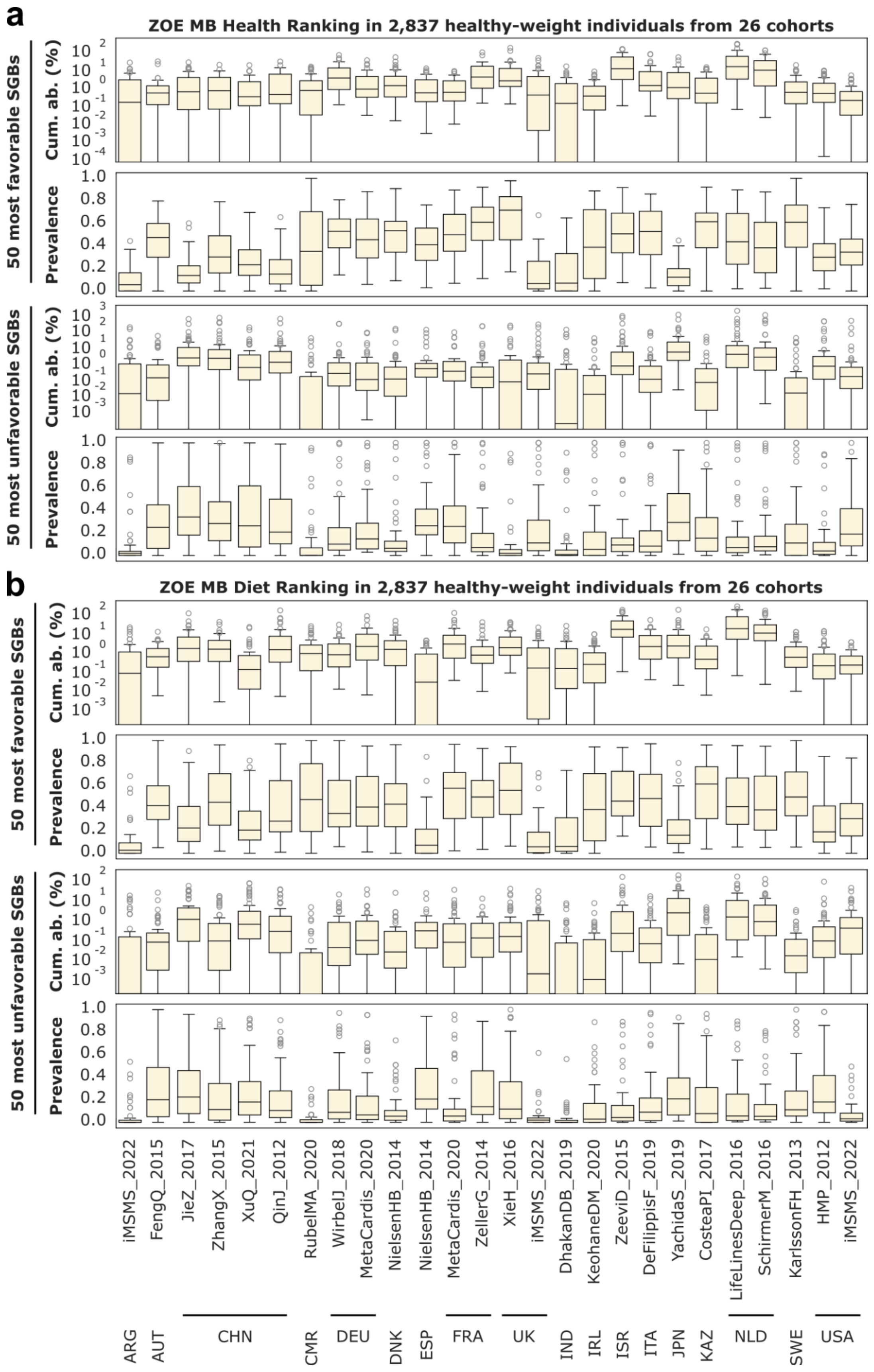

Supplementary Fig. 4. The distribution of the ZOE MB Health and Diet-ranked SGBs in 2,837 healthy-weight individuals from 18 countries and 26 public cohorts. a) The distributions of the cumulative relative abundance and prevalence of the 50 most favorable (upper) and unfavorable (lower) SGBs in the ZOE MB Health rankings, showing variable distributions across datasets and countries. **b)** The cumulative relative abundance and prevalence of the 50 most favorable (upper) and unfavorable (lower) SGBs in the ZOE MB Diet rankings. The effect of sequencing depth, which can be associated with datasets and countries for both analyses, is assessed in **Supplementary Table 10**.

Supplementary Fig. 5

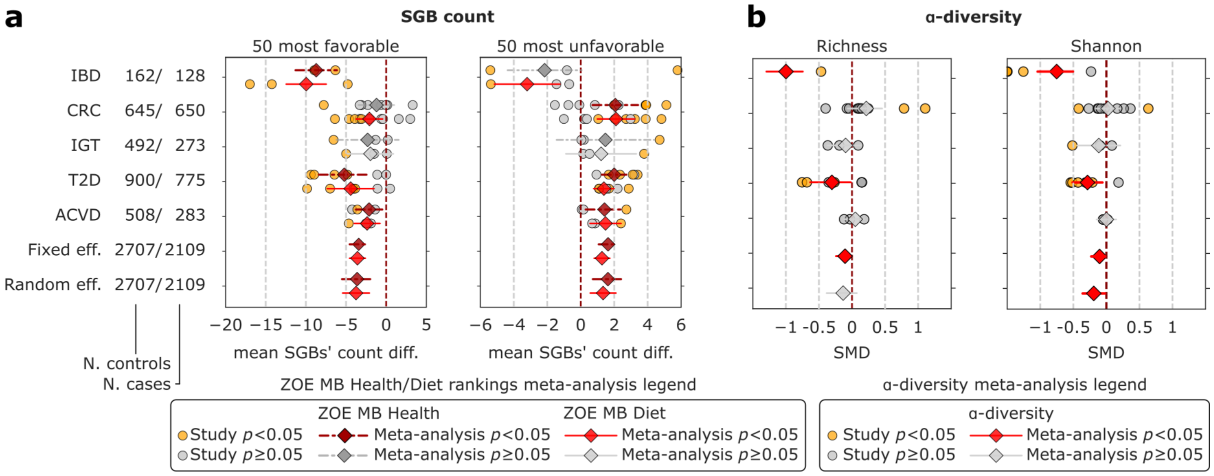

Supplementary Fig. 5. Meta-analyses of the 50 most favorable and unfavorable SGBs according to the ZOE MB Health and Diet-rankings across disease categories. a) Meta-analyses of the mean difference of the number of the 50 most favorable (left) and unfavorable (right) SGBs found in each sample from 25 public cohorts from five diseases (age ≥ 16 and BMI ≥ 18.5, retrieved from curatedMetagenomicData 3 [85]). Dark-red and light-red markers refer to ZOE MB Health and Diet ranks, respectively. Circles represent the mean count difference from a linear model adjusted by sex, age, and BMI. Diamonds indicate the coefficient of a random-effect meta-analysis (**Methods**). **b)** Meta-analysis on alpha-diversity (richness and Shannon's entropy) in discriminating between cases and controls. Error bars represent the 95% confidence interval.

Supplementary Table Legends

Table S1. Overview of the main characteristics of the PREDICT cohorts.

Table S2. Machine learning predictions via Random Forest (classification and regression) for personal, dietary, fasting, and postprandial cardiometabolic markers.

Table S3. Analysis of the consistency of the top predicted markers (AUC>0.7 and Spearman>0.4).

Table S4. Correlations of all markers across categories.

Table S5. The ZOE MB Health and Diet ranks by cohort, geography, and globally.

Table S6. Analysis of the consistency of the 50 most favorably and unfavorably ZOE MB Health-ranked SGBs.

Table S7. Taxonomic information of the 50 most favorably and unfavorably ZOE MB Health-ranked SGBs.

Table S8. Differently ranked SGBs between ZOE MB Health and ZOE MB Diet ranks.

Table S9. List of public datasets included in the BMI groups meta-analysis.

Table S10. Comparing the median of the 50 most favorable and unfavorable ZOE MB Health and Diet-ranked SGBs by BMI group.

Table S11. Country-wise comparison on healthy-weight individuals only for the 50 most favorable and unfavorable ZOE MB Health and Diet ranked SGBs.

Table S12. Meta-analyses on BMI categories considering the mean count difference of the 50 most favorable ZOE MB Health-ranked SGBs.

Table S13. Meta-analyses on BMI categories considering the difference in cumulative abundance of the 50 most favorable ZOE MB Health-ranked SGBs.

Table S14. Meta-analyses on BMI categories considering the mean count difference of the 50 most unfavorable ZOE MB Health-ranked SGBs.

Table S15. Meta-analyses on BMI categories considering the difference in cumulative abundance of the 50 most favorable ZOE MB Health-ranked SGBs.

Table S16. Meta-analyses on BMI categories considering the mean count difference of the 50 most unfavorable ZOE MB Diet-ranked SGBs.

Table S17. Meta-analyses on BMI categories considering the difference in cumulative abundance of the 50 most favorable ZOE MB Diet-ranked SGBs.

Table S18. BMI meta-analyses on the number of the 50 most unfavorable ZOE MB Diet-ranked SGBs.

Table S19. BMI meta-analyses on the cumulative relative abundance of the 50 most unfavorable ZOE MB Diet-ranked SGBs.

Table S20. List of public case-control datasets included in the meta-analysis.

Table S21. Case-control meta-analyses on the number of the 50 most favorable and unfavorable ZOE MB Health and Diet-ranked SGBs.

Table S22. Case-control meta-analyses on cumulative relative abundance of the 50 most favorable and unfavorable ZOE MB Health and Diet-ranked SGBs.

Table S23. Case-control meta-analyses on the total normalized ZOE MB Health and Diet ranks scores, weighted and unweighted by relative abundance.

Table S24. Case-control meta-analyses on alpha diversity (as Richness and Shannon entropy).

Table S25. Prevalent SGBs from the BIOME and METHOD cohorts with their mean relative abundance values and prevalence at both baseline and endpoint, the p-values and q-values from the Wilcoxon test and the FDR-BH multiple hypothesis testing correction, respectively. The ratio of the mean relative abundance at endpoint over baseline and its log2 transformation are reported, as well as the ZOE MB Health and Diet ranks, if the SGB was ranked.
