# Supplementary material for: Gut micro-organisms associated with health, nutrition and dietary interventions
Source: Nature. 2025 Dec 10;650(8101):450–8. doi: 10.1038/s41586-025-09854-7 (PMC12893911; doi:10.1038/s41586-025-09854-7)
Supplement: Supplementary file 2 — Reporting Summary [file 41586_2025_9854_MOESM2_ESM.pdf]

nature portfolio

Corresponding author(s): Francesco Asnicar, Nicola Segata

Last updated by author(s): Sep 30, 2025

Reporting Summary

Nature Portfolio wishes to improve the reproducibility of the work that we publish. This form provides structure for consistency and transparency in reporting. For further information on Nature Portfolio policies, see our Editorial Policies and the Editorial Policy Checklist.

Statistics

For all statistical analyses, confirm that the following items are present in the figure legend, table legend, main text, or Methods section.

n/a	Confirmed	
☐	☒	The exact sample size (*n*) for each experimental group/condition, given as a discrete number and unit of measurement
☐	☒	A statement on whether measurements were taken from distinct samples or whether the same sample was measured repeatedly
☐	☒	The statistical test(s) used AND whether they are one- or two-sided<br>*Only common tests should be described solely by name; describe more complex techniques in the Methods section.*
☐	☒	A description of all covariates tested
☐	☒	A description of any assumptions or corrections, such as tests of normality and adjustment for multiple comparisons
☐	☒	A full description of the statistical parameters including central tendency (e.g. means) or other basic estimates (e.g. regression coefficient) AND variation (e.g. standard deviation) or associated estimates of uncertainty (e.g. confidence intervals)
☐	☒	For null hypothesis testing, the test statistic (e.g. *F*, *t*, *r*) with confidence intervals, effect sizes, degrees of freedom and *P* value noted<br>*Give P values as exact values whenever suitable.*
☒	☐	For Bayesian analysis, information on the choice of priors and Markov chain Monte Carlo settings
☒	☐	For hierarchical and complex designs, identification of the appropriate level for tests and full reporting of outcomes
☐	☒	Estimates of effect sizes (e.g. Cohen's *d*, Pearson's *r*), indicating how they were calculated

Our web collection on statistics for biologists contains articles on many of the points above.

Software and code

Policy information about availability of computer code

Data collection	Sequenced metagenomic data were collected in FASTQ files, while individuals' metadata were self-reported using the ZOE mobile App.
Data analysis	MetaPhlAn (v4.beta.1 and v4.beta.2, both with database version Jan21_CHOCOPhlAnSGB_202103). All analysis were carried out in Python 3 (v3.12.0) using the following libraries: numpy (v1.26.2), scipy (v1.11.4), pandas (v2.1.3), pingouin (v0.5.4), sklearn (v1.3.2), and statsmodels (v0.14.0), and visualization was performed using the matplotlib (v3.8.2) and seaborn libraries (v0.11.2). Public metagenomic cohorts were retrieve from curatedMetagenomicData (v3). The custom Python code developed for the meta-analyses performed on public data and included in this work is available in the Github repository: https://github.com/SegataLab/inverse_var_weight and Zenodo (https://doi.org/10.5281/zenodo.17236262). The MetaPhlAn code for the taxonomic profiling is available in the Github repository: https://github.com/biobakery/MetaPhlAn, on Zenodo (https://doi.org/10.5281/zenodo.17236262), and via bioconda at https://bioconda.github.io/recipes/metaphlan/README.html.

For manuscripts utilizing custom algorithms or software that are central to the research but not yet described in published literature, software must be made available to editors and reviewers. We strongly encourage code deposition in a community repository (e.g. GitHub). See the Nature Portfolio guidelines for submitting code & software for further information.

Data

Policy information about availability of data

All manuscripts must include a data availability statement. This statement should provide the following information, where applicable:

- Accession codes, unique identifiers, or web links for publicly available datasets
- A description of any restrictions on data availability
- For clinical datasets or third party data, please ensure that the statement adheres to our policy

The human genome version used in the preprocessing of the microbiome samples is the GRCh37 genome assembly (hg19, GCA_000001405.1). Raw metagenomic samples, along with metadata information (sex, age, BMI, and country) and microbiome profiles for all participants of the ZOE PREDICT Studies, are publicly available. Metagenomes from PREDICT 1 are publicly available as previously reported 9, while the PREDICT 2 and PREDICT 3 cohorts (US21, US22A, and UK22A) are deposited in the European Nucleotide Archive (ENA) of the European Bioinformatics Institute (EBI) under accession numbers PRJEB75460, PRJEB75462, PRJEB75463, and PRJEB75464, and are publicly accessible. Sex, age, BMI, country, and quantitative taxonomic profiles for each sample are publicly available within the curatedMetagenomicData package 60 and at Zenodo (https://doi.org/10.5281/zenodo.15308000). The full list of species for the ZOE Microbiome Rankings are publicly available at https://zoe.com/our-science/microbiome-ranking, where future updates will also be made available. The version of the ZOE Microbiome Rankings discussed in the present work is reported in Supplementary Table 5. To protect participant privacy, individual participant clinical data are not publicly available and cannot be deposited in public repositories. ZOE is committed to supporting scientific reproducibility, and researchers requesting access to the data required for this purpose may submit a research proposal. Proposals, researchers, or institutions requesting data for reproducibility will be approved if they meet the standard criteria related to ethics, privacy, and data protection regulations. For reproducibility of the whole non-parametric analysis used to define the rankings, the required host parameters will be provided as ordered data points. This data is deposited in Zenodo (https://doi.org/10.5281/zenodo.17236383) and is encrypted; access to the data will be granted to researchers whose proposals will be approved. For proposals not intended for reproducibility, ZOE reserves all the rights to evaluate the scientific priority and relevance of the request. Researchers can request a proposal form by emailing data.papers@joinzoe.com. All proposals will be reviewed by a sub-panel of the ZOE Scientific Advisory Board within four working weeks. Approved researchers will be required to enter into a data-sharing agreement with ZOE. All data from non-PREDICT external public cohorts used to validate the rankings are available in full at https://doi.org/10.5281/zenodo.17236262.

Research involving human participants, their data, or biological material

Policy information about studies with human participants or human data. See also policy information about sex, gender (identity/presentation), and sexual orientation and race, ethnicity and racism.

Reporting on sex and gender	Sex information (not gender) were self-reported by individuals through the ZOE App with informed consent. Sex was used in the analysis as a covariate as detailed in the Methods.
Reporting on race, ethnicity, or other socially relevant groupings	Data on socially relevant variables such as race or ethnicity were not considered in the analyses.
Population characteristics	Overall, participants were aged around 50 years old, with about 80% females.
Recruitment	Information pertaining to the publicly available datasets used in this work are available from their respective publications: Asnicar F, Nat Med, 2021 (PREDICT 1, https://doi.org/10.1038/s41591-020-01183-8), Bermingham KM, Nat Med, 2024 (METHOD, https://doi.org/10.1038/s41591-024-02951-6), and Creedon AC, bioRxiv, 2024 (BIOME, https://doi.org/10.1101/2024.07.02.24309816). The other ZOE PREDICT studies have the following protocol IDs: METHOD (NCT05273268), BIOME (NCT06231706), PREDICT 2 (NCT03983733), PREDICT 3 US 21 and 22A (IRB Pro00044316), PREDICT3 UK 22A and 23ART (HR-23/24-28300). All PREDICT 3 cohorts refers to the Clinicaltrials.gov identifier: NCT04735835. Participants from the ZOE PREDICT studies paid a private company (ZOE Ldt.) to sequence their microbiomes and filled out informed consent forms that allows the usage of their data by ZOE for scientific purposes.
Ethics oversight	All study protocols are registered on clinicaltrials.gov and procedures are compliant with all relevant ethical regulations. Ethical approval for the PREDICT 1 study was obtained in the United Kingdom from the King's College London Research Ethics Committee (REC) and Integrated Research Application System (IRAS 236407), and in the United States from the institutional review board (Partners Healthcare Institutional Review Board (IRB) 2018P002078). Ethical approval for the PREDICT 2 study (Pro00033432) was obtained from Advarra IRB. Ethical approval for the PREDICT 3 study (Pro00044316, HR/DP-21/22-28300 and HR/DP-24/25-45829) was obtained from Advarra IRB and King's College London REC. Ethical approval for the METHOD study (Pro00044316; protocol no. 00044316) was obtained from Advarra IRB. Ethical approval for the BIOME study (HR/DP-23/24-39673) was obtained through King's College London REC. All participants provided written informed consent and all studies were carried out in accordance with the Declaration of Helsinki and Good Clinical Practice.

Note that full information on the approval of the study protocol must also be provided in the manuscript.

Field-specific reporting

Please select the one below that is the best fit for your research. If you are not sure, read the appropriate sections before making your selection.

☒ Life sciences ☐ Behavioural & social sciences ☐ Ecological, evolutionary & environmental sciences

For a reference copy of the document with all sections, see nature.com/documents/nr-reporting-summary-flat.pdf

Life sciences study design

All studies must disclose on these points even when the disclosure is negative.

Sample size	No a priori sample size calculation was performed, as we gather and analyzed 34,694 microbiome samples, leveraging the largest (to the best of our knowledge) and most comprehensive microbiome cohorts, providing robust statistical power sufficient for the primary exploratory and hypothesis-generating objectives of this study.
Data exclusions	Only one individual from the PREDICT 2 cohort was excluded from all the analysis as it had an empty (no species detected) microbiome profile.
Replication	The findings were assessed for reproducibility and validated across multiple independent cohorts, including publicly available external ones. This rigorous validation, achieved through the analysis of distinct datasets spanning different geographies and populations, substantiates the robustness of the defined microbial species ranks. No results/experiments replications were performed.
Randomization	Randomization was necessary and done with the machine learning analyses, in which a ten-times, ten-folds cross-validation was performed.
Blinding	All microbiome analyses were carried out blinded to group allocation and subject metadata by the primary analyst. Sample extraction and sequencing were performed independently by the processing laboratory, which was also separate from ZOE Ltd. employees. The use of distinct, independent parties for sample processing, sequencing, and final statistical analysis inherently minimized the potential for systematic bias in the analytical pipeline.

Reporting for specific materials, systems and methods

We require information from authors about some types of materials, experimental systems and methods used in many studies. Here, indicate whether each material, system or method listed is relevant to your study. If you are not sure if a list item applies to your research, read the appropriate section before selecting a response.

Materials & experimental systems

n/a	Involved in the study
☒	☐ Antibodies
☒	☐ Eukaryotic cell lines
☒	☐ Palaeontology and archaeology
☒	☐ Animals and other organisms
☒	☐ Clinical data
☒	☐ Dual use research of concern
☒	☐ Plants

Methods

n/a	Involved in the study
☒	☐ ChIP-seq
☒	☐ Flow cytometry
☒	☐ MRI-based neuroimaging

Plants

Seed stocks	n/a
Novel plant genotypes	n/a
Authentication	n/a
